# Binding free energy predictions in host-guest systems using Autodock4. A retrospective analysis on SAMPL6, SAMPL7 and SAMPL8 challenges

**DOI:** 10.1007/s10822-021-00388-4

**Published:** 2021-05-24

**Authors:** Lorenzo Casbarra, Piero Procacci

**Affiliations:** University of Florence, Department of Chemistry, Via Lastruccia n. 3, I-50019 Sesto Fiorentino FI, Italy

**Keywords:** SAMPL7, Binding free energy, Non-equilibrium, Crooks theorem, Fast switching, Hamiltonian replica exchange, HREX, Solute tempering, Torsional tempering

## Abstract

We systematically tested the Autodock4 docking program for absolute binding free energy predictions using the host-guest systems from the recent SAMPL6, SAMPL7 and SAMPL8 challenges. We found that Autodock4 behaves surprisingly well, outperforming in many instances expensive molecular dynamics or quantum chemistry techniques, with an extremely favorable benefit-cost ratio. Some interesting features of Autodock4 predictions are revealed, yielding valuable hints on the overall reliability of docking screening campaigns in drug discovery projects.

**Supplementary Information:**

The online version contains supplementary material available at 10.1007/s10822-021-00388-4.

## Introduction

SAMPL (Statistical Assessment of the Modeling of Proteins and Ligands) [1–5] are NIH-funded community-wide blind challenges for advancing computational methodologies as predictive tools in rational drug design. The challenges were started in 2010 and are organized on a quasi-yearly basis, with the SAMPL8 deadline set at February 2021. SAMPL challenges focus on the determination of the absolute binding free energy (ABFE) in host-guest systems involving hosts such as cyclodextrines [6], Cucurbituryl-like [7] and Octa-acids [8] cavitands, and drug-like small molecule compounds (SMC), as well as on physical properties of SMCs such as solvation free energies, pKa, LogP, and LogD.

**Fig. 1:**
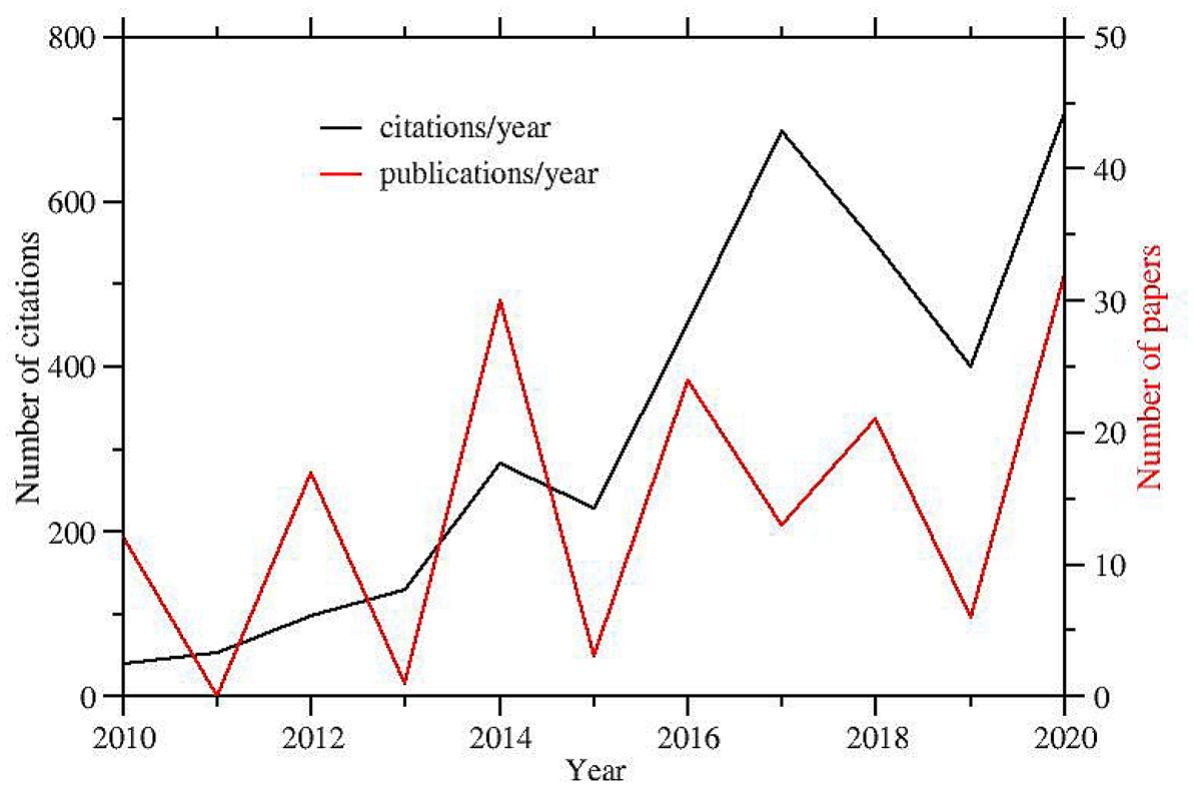
Publications per year (right scale) on SAMPL challenges and corresponding citations per year (left scale). Data taken from the Scopus database (www.scopus.com)

The SAMPL initiative has attracted widespread attention in the drug design scientific community. In the last decade, $\simeq$160 papers dealing with SAMPL predictions have been published on drug design oriented journals with a constant increase of the citation rate (see Fig. 1)

In the challenges, disparate methodologies are assessed, from quantum chemistry (QM) approaches or Molecular Dynamics (MD) computational strategies to semiempirical data-driven protocols. In many instances, submitted predictions for host-guest ABFE’s are produced using sophisticated and computationally demanding MD-based methodologies such as alchemical free energy perturbation [9–11], potential of mean force along physical host-guest coordinates [12], and nonequilibrium alchemy [10, 11] or QM-based high-level techniques using implicit solvation models [13] or QM/MM Hamiltonians.

While molecular docking has been often used by participants in the preparatory stages for pose assessment or identification, this technique has been very rarely used in the SAMPL challenges like the one and only tool for predicting ABFE’s [14]. Indeed, accurate binding free energies are universally believed beyond the capabilities of docking scoring functions. The docking paradigm relies in fact on important approximations, such as implicit solvent, rigid (or mostly rigid) receptor, crude estimates of the entropy gain or loss upon binding, absence of microsolvation contributions due to explicit water molecules.

Recent analysis on drug-protein systems based on binary classification [15, 16] have shown that modern commercial or freely available docking programs like Autodock [17], Idock [18] and Glide [19] yields a median area under the receiver operating characteristic curve (ROC-AUC) of $\simeq$ 0.70 on well established drug-receptor benchmark sets such as DUD-E [20]. This value indicates that docking has an average probability of discerning active from inactive compounds (decoys) only 40% higher than that based on the flipping a coin. Despite these modest performances, docking techniques are commonly used in drug discovery. Docking based approaches, for example, account for nearly 6% of all peer-reviewed Covid-19-related scientific output in 2020 according to the Scopus database. Such widespread usage in drug design is due to the remarkable efficiency of this method in comparison to more rigorous MD-based or QM-based physical approaches. A single node of the Summit high performing computer (HPC) at the Oak Ridge National Laboratory can deliver in 24 hours the docking scoring functions of 250000 compounds on Covid-19-related targets with full structural optimization of the ligand [21]. On similar facilities, an efficient MD-based technology can require several days to compute the absolute binding free energies of few tens of host-guest pairs in a typical SAMPL challenge [22].

Due to its efficiency, docking is routinely being used as a triaging tool for identifying potential ligands of important biological targets such as the SARS-CoV-2 proteinase [23, 24], to be further assessed using seemingly more accurate and far more computationally-demanding approaches. It is therefore of interest to rigorously evaluate the predictive performance of molecular docking in the SAMPL challenges for host-guest ABFE’s, albeit in retrospective. While in some of the past SAMPL challenges molecular docking was rarely tested [14] or used to produce the reference *null* model [4], to our knowledge such systematic assessment by way the typical SAMPL metrics (correlation coefficients, mean unsigned errors, Kendall coefficient, etc. ) has not been undertaken yet. To this end, we have computed, using a popular and widely available docking program, Autodock4 [17], the ABFE for all host-guest pairs taken from the three *latest* SAMPL6 to SAMPL8 challenges, with the idea that the lessons learned in SAMPL1-SAMPL5 challenges afforded a tuning or optimization of the most used advanced methodologies for ABFE predictions. Results were indeed surprising. Autodock4 did in general quite well, over-performing costly and complex technologies in many instances. Some interesting features of docking predictions are revealed, yielding valuable hints on the overall reliability of docking screening campaigns.

The paper is organized as follows. In section “Methods” we succinctly provide the main ingredients and technical details of host-guest docking calculations. In the “Data processing” section, we describe the content of the archive provided as supporting information, including data and application software for straightforwardly reproducing our results. Autodock4 predictions are presented in the “Results sections” along with a bird’s eye survey of the SAMPL6-SAMPL8 challenges. Finally in the last section, we draw some concluding remarks.

## Methods

In molecular docking, host-guest or drug-receptor *scoring functions* are generally computed using simplified interaction potentials based on pairwise atom-atom interactions supplemented with entropy-related desolvation/conformational terms. These functions represent the ABFE for the docked complex as a sum of various contributions, relying on empirical parameters often refined or trained through knowledge-based approaches [25].The Autodock4 code uses [17] a scoring function of the kind

1 $$\Delta G_{{bind}} {\text{ }} = {\text{ }}W_{{vdw}} \Delta G_{{vdw}} + W_{{elec}} \Delta G_{{elec}} + W_{{hbond}} \Delta G_{{hbond}} + W_{{desolv}} \Delta G_{{desolv}} + \Delta G_{{conf}}$$

where $\varDelta G_{\rm {vdw}}$ and $\varDelta G_{\rm {elec}}$ are due to the atom-atom 12-6 Lennard-Jones potentials and Coulomb charge-charge interactions with distance-dependent dielectric screening, respectively, $\varDelta G_{\rm {hbond}}$ is computed using a directional potential accounting for H-bond interactions, $\varDelta G_{\rm {desolv}}$ is a term representing the solvation free energy change upon binding, and $\varDelta G_{\rm {conf}}$ is related to the entropy loss of the ligand upon binding. The weighting constants *W* in Eq. 1 are optimized (trained) to calibrate the empirical free energy based on a set of experimentally determined binding constants. Explicit expressions of the $\varDelta G$ contributions in Eq. 1 in terms of pairwise interactions are given in Ref. [17].

In the last decade, most of commercial and publicly available docking approaches have evolved towards the calibration of efficient scoring functions using machine learning (ML) techniques, by removing, rather than adding, “physical” components [26]. Autodock4, for examples, in modeling electrostatic interactions, uses distance dependent dielectric screening rather than more rigorous (and much more expensive) Poisson-Boltzmann or Generalized Born approaches. In the Vina program [27], a popular and *faster* alternative of the Autodock4 code, atomic charges are no longer included in the scoring functions, whose electrostatics is described only by directional h-bonds terms.

Molecular docking with Autodock4 starts with the calculation, performed by the Autogrid4 program [17], of a grid potential (in some user-defined region of interest) due to the atoms of the *rigid* macromolecule (host in our case). Actual docking of the fully flexible ligand reduces hence to a global minimization process of the function Eq. 1 with respect to the *ligand* coordinates only, relying on the previously determined grid potential. Flexible residues/groups of the receptor/host do not contribute to the grid potential and they are *de facto* considered as a “ligand” appendix in the docking minimization process, thereby expanding the docking minimization cost.

In the present study, docking calculations were run on the configurations of the hosts and guests provided in the .sdf files downloaded from the officials SAMPL6 and SAMPL7 and SAMPL8 GitHub repositories [28]. The hosts in these challenges include Cucurbituril cavitands [29], the Triptycene walled glycoluril trimer [7], various mono-3-substituted $\beta$-cyclodextrin analogues [6], and the Gibb Deep Cavity Cavitands or Octa-acids [8]. The guests are small molecule compounds with molecular weight (MW) comprised in the range $90 \le \rm {MW} \le 510$ Da. In the Table 1, we report detailed information on the challenges

**Table 1:** The host types and the number of ligands are given for each challenges

	OA	TEMOA	exoOA	CB8	CLIP	CD
SAMPL6	8 (45)	8 (45)	n/a	14(38)	n/a	n/a
SAMPL7	8 (16)	n/a	8(16)	n/a	16(8)	16(7)
SAMPL8	n/a	n/a	n/a	7(35)	n/a	n/a

In parenthesis we report the number of submissions (ranked or not ranked) for each system. CB8: cucurbituril); OA (octa-acid); TEMOA: tem-octa-acid; exoOA: exo octa-acid; CD: beta-cyclodextrin derivatives); CLIP: open cucurbituril-like cavitand

The chemical structures of all guests and hosts can be found in the cited GitHub repositories [28] as well as in the provided SI. On overall, we calculated the ABFE for 82 host-guest systems.

In all cases, we used the Autogrid4 default settings for grid generation with the hosts being considered as *rigid*. More in detail, the docking region is a cubic box of side-length of 15 Å with a grid spacing in each direction of 0.375 Å, centered at the host center of mass. The .sdf files, prior of being fed to Autodock4, were converted into .pdb files using OpenBabel [30] specifying, via the -p option, the pH used in the SAMPL experiments. [28] Prediction files submitted by all SAMPL participants as well as experimental data were also downloaded from the cited GitHub repositories and stored in the Supporting Information (SI).

Quality metrics for our Autodock4 prediction and for all other submissions (including Vina) were obtained using the scripts in the compressed archive provided as SI. The archive contains all input/out generated by the Autogrid4 or Autodock4 programs on the SAMPL6 to SAMPL8 challenges, as well as the application scripts (with essential documentation) for data processing. For a detailed description of the SI archive, see Section “Data processing” further below.

Autodock4 performs a cluster analysis or “structure binning” [17] based on all-atom root mean square deviation (RMSD), ranking the resulting families of docked conformations in order of increasing binding free energies, as computed according to Eq. 1. For highly symmetric hosts, such as the cucurbituril or the octa-acids cavitands in SAMPL6 SAMPL7 and SAMPL8, or for C1-symmetry compounds with highly symmetric binding cores such as the beta-cyclodextrin derivatives in SAMPL7, the RMSD-categorized docking families are considered as *competitive binding poses* [31] or *symmetry-related poses* [32]. In both cases, we have estimated the ABFE as

2 $$\begin{aligned} \varDelta G = -RT \ln \left( \sum _i e^{-\beta \varDelta G_i} \right) \end{aligned}$$

where $\varDelta G_i$ refer to the final ranked free energies in the .dlg Autodock4 output file. Docking calculations on the 82 host-guest systems required less than one hour on a low-end 8-processor CPU workstation.

## Data processing

**Fig. 2:**
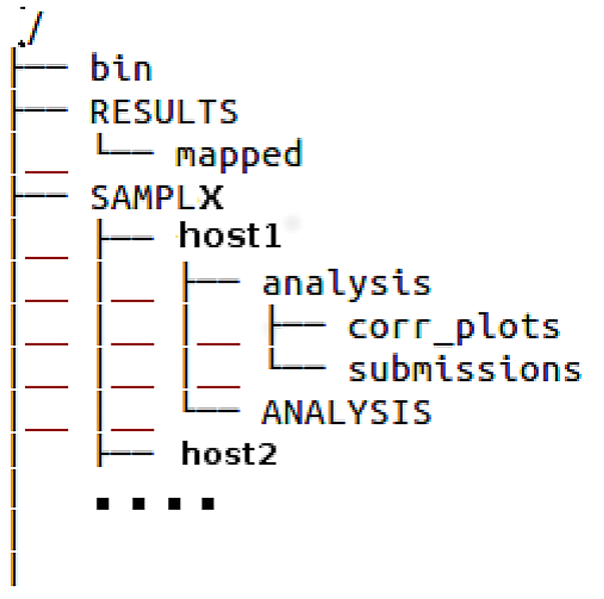
Directory tree of the workspace directory generated from the SI archive

The compressed archive provided as SI.zip, when unzipped, generates a directory called workspace. The directory tree of the workspace directory is shown in Fig. 2. The workspace directory contains the following sub-directories:

bin: includes application scripts for data processing. These commands are activated, under any unix operating system, by sourcing the file source_this_file.bash in this directory. Detailed information for executing these scripts can also be found in the README file inside this directory.

RESULTS: contains the results of all submissions (Autodock4 included) for the ABFE’s of all host-guest systems in the SAMPL6, SAMPL7, SAMPL8 challenges.

SAMPLX (where X=6,7,8) : Each of these three directories contains a number of sub-directories corresponding to the hosts used the in the challenge. In each host sub-directory, the input/output Autodock4 files are stored. Results can be replicated using the docking.bash script provided in the bin directory. Autodock4 and MGLtools must be installed before executing the docking.bash. Installation instructions are given in the docking.bash file.

Each of the host sub-directories contains the ANALYSIS and analysis sub-directory. ANALYSIS contains all the *original* submissions files (taken from the GitHub site [28]) for the corresponding SAMPLX-host challenge. From the analysis directory, data metrics for all ANALYSIS submissions can be produced by issuing the command analysis.bash provided in the bin directory. For more information see the README file and the comments in the analysis.bash script in the bin directory. The files predictions_from_perl.names lists the method (as specified by the participants) used in the corresponding SAMPLX-host challenge. This file can be generated using the perl script samplmanager.pl (see Documentation in the bin directory).

## Results

**Fig. 3:**
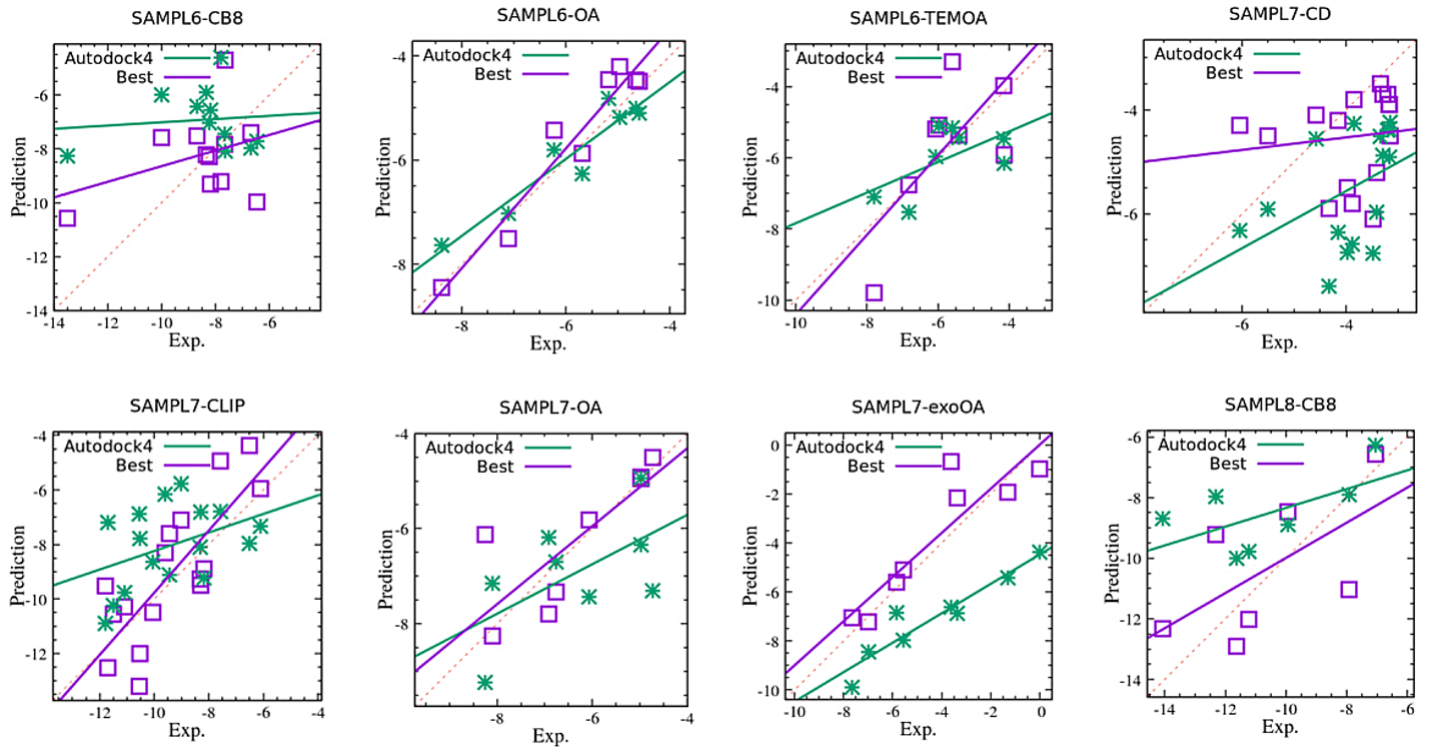
Correlation plot experimental *vs* computed binding free energies (in kcal/mol) for the Autodock prediction set (green) and the best (MAE) prediction set (magenta) in host-guest systems included in the the SAMPL6, SAMPL7 and SAMPL8 challenges. The challenges are identified by the acronym SAMPL*x*-*host*, where $x=6,7,8$ and *host* is CB8 (cucurbituril), OA (octa-acid), TEMOA (tem-octa-acid), exoOA (exo octa-acid), CD (beta-cyclodextrin derivatives), CLIP (open cucurbituril-like cavitand)

In the correlation plots reported in Fig. 3 we compare the results obtained with Autodock4 to the best prediction set in the SAMPL6,SAMPL7, and SAMPL8 challenges.

We use the mean absolute error (MAE) for ranking the best submissions. This quantity is less sensitive to outliers than the root mean square deviation or correlation coefficients are. While The Pearson and Kendall coefficients, $\rho$ and $\tau$, are related to *precision* and *reproducibility* , MAE is a direct measure of the *accuracy* of a methodology, i.e. it expresses the mean closeness of the predicted value to the the experimental value. Methods yielding data with acceptable or good Pearson correlation coefficient and large MAE are likely to be affected by an undetected systematic bias, a serious drawback in a blind prediction for *absolute* binding free energies.

Figure 3 shows that Autodock4 predictions, quite expectedly, are systematically worse than the corresponding *best* prediction set. In one case, SAMPL7-CD, Autodock4, while being better correlated to the experimental data, exhibits an MAE that is 70% larger than that of the best prediction set. Results are further detailed in Table 2. Among the top-performing approaches, we consistently find MD-based

**Table 2:** Quality metrics for the Autodock predictions (AD) sets and best predictions (best). MAE, $\rho$, and $\tau$ refer to mean absolute error (in kcal/mol), the Pearson correlation coefficient, and the Kendall and coefficient

	MAE		$\rho$		$\tau$		
Challenge	AD	Best	AD	Best	AD	Best	Method
SAMPL6-CB8	2.10	1.51	0.10	0.36	− 0.24	0.09	MD/DDM/GAFF [33]
SAMPL6-OA	0.41	0.40	0.95	0.96	0.64	0.57	MD/PMF/GAFF$^a$
SAMPL6-TEMOA	0.77	1.03	0.58	0.95	0.14	0.79	MD/PMF/CGenFF$^b$
SAMPL7-CD	1.60	1.04	0.43	0.12	0.40	0.21	MD/FS/GAFF [34]
SAMPL7-CLIP	1.82	1.39	0.34	0.79	0.28	0.60	MD/DDM/AMOEBA [35]
SAMPL7-OA	1.00	0.54	0.59	0.80	0.25	0.75	MIXED [36]
SAMPL7-exoOA	2.76	0.92	0.95	0.90	0.79	0.71	MD/DDM/AMOEBA [35]
SAMPL8-CB8	2.08	1.71	0.60	0.65	0.43	0.52	MD/LGFE/CGenFF$^c$

The “Method” entry refers to the methodology used in the best prediction set (see text)

$^a$ See the SAMPL6-OA submission file finzb-973-OA-submission-19.txt in the SI

$^b$ See SAMPL6-TEMOA submission file vq30p-973-TEMOA-NHLBI-1.txt in the SI

$^c$ See SAMPL8-CB8 submission file CB8_SILCS_reweightedLGFE.txt in the SI

techniques, with the alchemical variants [10, 11], DDM (double decoupling method) or FS (fast switching), appearing in four of the top-performing cases, and with the Umbrella sampling/potential of mean force (PMF) approach [37] in two cases. In only one case (SAMPL7-OA), an ML mixed approach resulted as the top-performing method using MAE as metrics. This “victory”, however, was not confirmed in the parent SAMPL7-exoOA challenge where the mixed-ML protocol yielded a disappointing MAE of 2.55 kcal/mol. Concerning the force fields, the CHARMM generalized force field (CGenFF [38]) and the generalized AMBER force field (GAFF [39]) were used in two and four cases, respectively, in the top MD-based performing methods. The polarizable force field AMOEBA [40], in combination with the DDM alchemical method, was very successful in the SAMPL7 challenge. Quite consistently, QM based approaches are never found among the top-performing sets. Overall, the data indicate that the SAMPL challenges have failed so far to clearly identify the “best” methodology for ABFE prediction in the host-guest systems. MD-based results seem to strongly depend on the ability of the force field to deal with the systems under scrutiny and/or to the adopted simulation protocol.

**Table 3:** Autodock4 ranking in the SAMPL challenges

Challenge	MAE	$\rho$	$\tau$	*n*
SAMPL6-CB8	4	30	36	38
SAMPL6-OA	2	6	12	45
SAMPL6-TEMOA	1	19	35	45
SAMPL7-CD	5	1	1	8
SAMPL7-CLIP	3	4	4	8
SAMPL7-OA	4	4	8	15
SAMPL7-EOA	6	3	4	15
SAMPL8-CB8	3	17	11	35

MAE $\rho$, $\tau$ and *n* refer to the mean absolute error (kcal/mol), the Pearson correlation coefficient, the Kendall rank coefficient and the total number of submissions, respectively

Autodock4 in many instances is found to outperforms expensive MD-based or QM-based computational techniques used in the SAMPL challenges. In Table 3 we report the Autodock4 ranking for the MAE, $\rho$ and $\tau$ metrics obtained in the challenges. Interestingly, Autodock4 yields better MAE’s than correlation coefficients. This is, to some extent, a surprising result as the performances of docking scoring functions are usually measured on their ability to *rank* the ligands in the correct order rather than on accuracy. In this respect, Autodock4 has a probability of 75%, 60%, and 49% of being among the top-performing methods as far as MAE, $\rho$ and $\tau$ are concerned, respectively.

**Table 4:** Vina ranking in the SAMPL challenges

Challenge	MAE	$\rho$	$\tau$	*n*
SAMPL6-CB8	5 (− 1)	37 (− 7)	39 (− 3)	38
SAMPL6-OA	10 (− 8)	1 ( 5)	17 (− 5)	45
SAMPL6-TEMOA	11 (− 10)	42 (− 23)	39 (− 4)	45
SAMPL7-CD	5 ( 0)	2 (− 1)	2 (− 1)	8
SAMPL7-CLIP	6 (− 2)	9 (− 2)	7 ( 0)	8
SAMPL7-OA	5 (− 1)	1 ( 3)	4 ( 4)	15
SAMPL7-EOA	7 (− 1)	14 (− 11)	14 (− 10)	15
SAMPL8-CB8	10 (− 7)	29 (− 12)	29 (− 18)	35

MAE $\rho$, $\tau$ and *n* refer to the mean absolute error (kcal/mol), the Pearson correlation coefficient, the Kendall rank coefficient and the total number of submissions, respectively. In parenthesis we report the difference with respect to Autodock4 ranking

We have also tested the Vina1.1.2 docking program [27]. Vina, a derivation of Autodock4, uses a quite different scoring function based on Van der Waals surface distances (rather than internuclear as in Autodock4) with pair hydrophobic, repulsion, H-bond terms and rotatable bond penalties with empirically determined weights based on extensive ligand-protein data-sets. Unlike in Autodock4, no atomic charges are used in the Vina scoring functions [27]. Vina significantly improves the average accuracy of the binding mode predictions compared to AutoDock4, and it was found to be a strong competitor against popular commercial programs, resulting at the top of the pack in many cases [27]. In Vina, the calculation of grid maps and the assignment of atomic charges is not required. To launch a Vina docking run, besides the pdbqt structures of ligand and receptor, only the binding site position (the COM of the hosts in all cases) needs to be specified along with the size of the search cubic box. For the latter, we used a side-length of 15 Å  as for Autodock4. Rankings obtained with Vina in the SAMPL challenges are reported in Table 4. Vina turned out to be significantly less performing for ABFEs in host-guest systems than Autodock. Apparently, the *less physical* Vina empirical scoring functions, specifically trained on extensive databases of *ligand-receptor* systems, show some weaknesses in these kind of simple complexes.

**Fig. 4:**
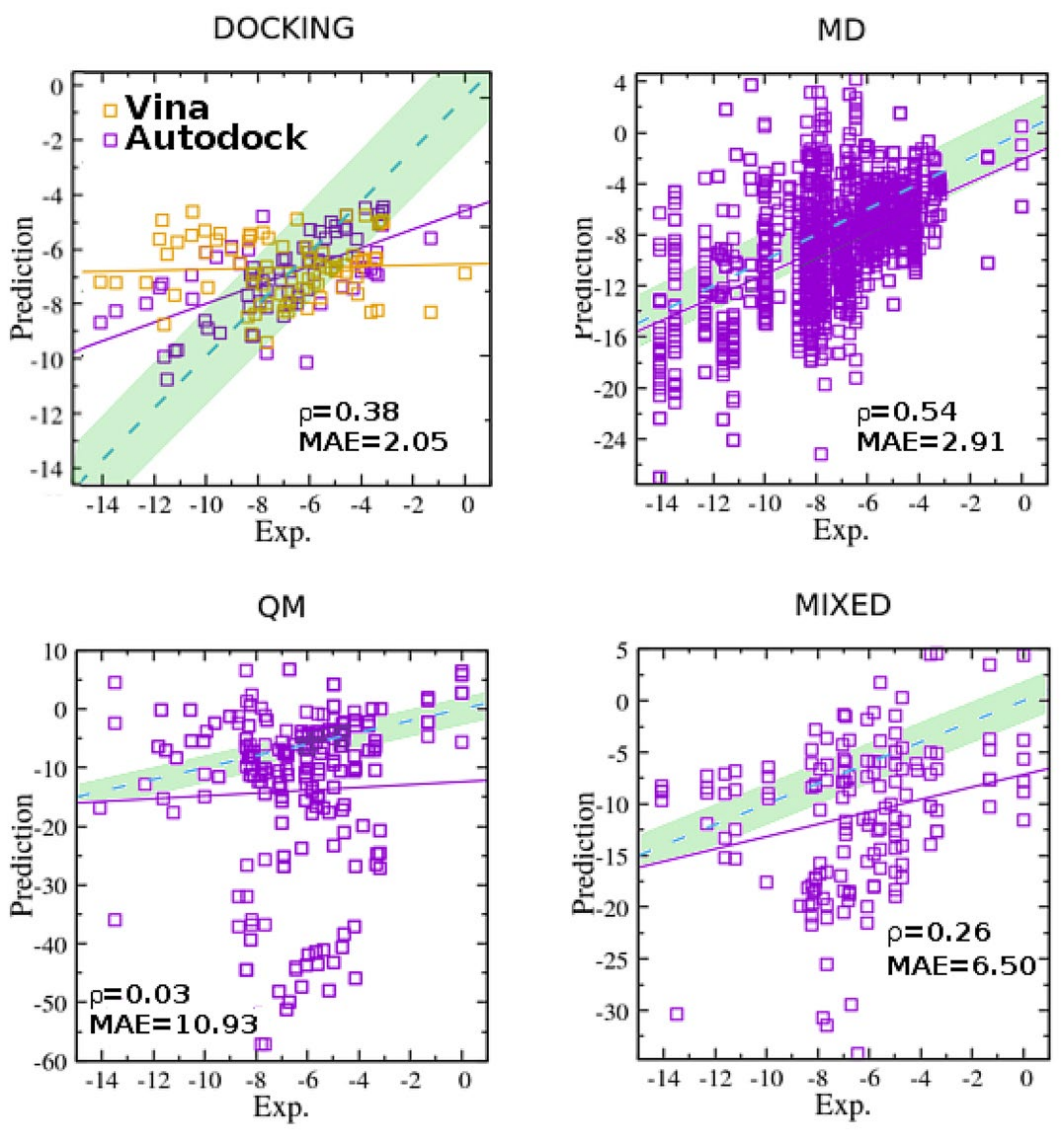
Correlation plots between experimental and calculated (in kcal/mol) host-guest binding free energies by category in the SAMPL6, SAMPL7, and SAMPL8 challenges. The violet solid and blue dashed line mark the best fitting line and perfect correlation, respectively. All points within the green-shaded area differ by less than 2 kcal/mol from the corresponding experimental data. The Docking panel includes data from Autodock4 and Vina

In Fig. 4, we report the correlation plots between experimental and predicted binding free energies by category. Docking data are represented by the Autodock4 and Vina prediction sets. The number of points in the MD, QM, and MIXED plots are indicative of the frequency with which the corresponding category has been adopted by the SAMPL participants. The MD-based methodologies are found to be the best correlated as measured by both the Pearson correlation coefficient $\rho$, while docking exhibit the lowest mean unsigned error MAE. QM and MIXED approaches yield, on the overall, the worst result.

From a drug-design perspective, the potential loss in economic value due to false negative is impossible to assess. False-negative are unavoidable in high-throughput screening processes (HTS), performed both experimentally and *in silico*. False positives, on the other hand, are one of the factors that currently restricts the discovery potential of HTS techniques, as they require time, energy, and high cost to be identified in wet-lab low-throughput protocols by medicinal chemists [41]. In this regard, a well established picture for assessing the capability of discerning active binders (true positive) from false positives (or false alarms) is that based on the *binary* metrics expressed by the receiver operating characteristics (ROC) graph [42]. Given a prediction method (or *classifier*), the ROC curve is constructed by assuming that ligands can be clumped in two groups, namely good or bad binders (*p* instances and *n* instances, respectively) according to some threshold ABFE value *t*. Below *t* and above *t*, ligand are good binders and bad binders, respectively. By continuously varying the threshold (starting from a very stringent (i.e. low) value of t), for each *t*, the points on the ROC curve can be constructed from the correlation data by grouping the outcomes into the “false positives” (*fp*) when according to the classifier (e.g docking or MD) the ABFE is below the given threshold *t* (good binder) while the experimental value (or instance) is above *t* (bad binder), and into “true positives” (*tp*), when the classifier and the experimental instance are both indicating a good binder. The false positive rate (FPR) and true positive rate (TPR) are given by $\rm {FPR}= fp/n$ and $\rm {TPR}= tp/p$. The lower left point (FPR=0,TRP=0) in the ROC square represents the strategy of never issuing a a good binder, and is obtained with the possible most stringent threshold *t* (no true positive of false positive: all outcomes are in the non-binder group). The opposite strategy, of unconditionally issuing good binder classifications, is represented by the upper right point (FPR=1,TRP=1). In the SI, we provide a simple awk script (roc.awk) to compute the ROC curve form a set of correlation data.

**Fig. 5:**
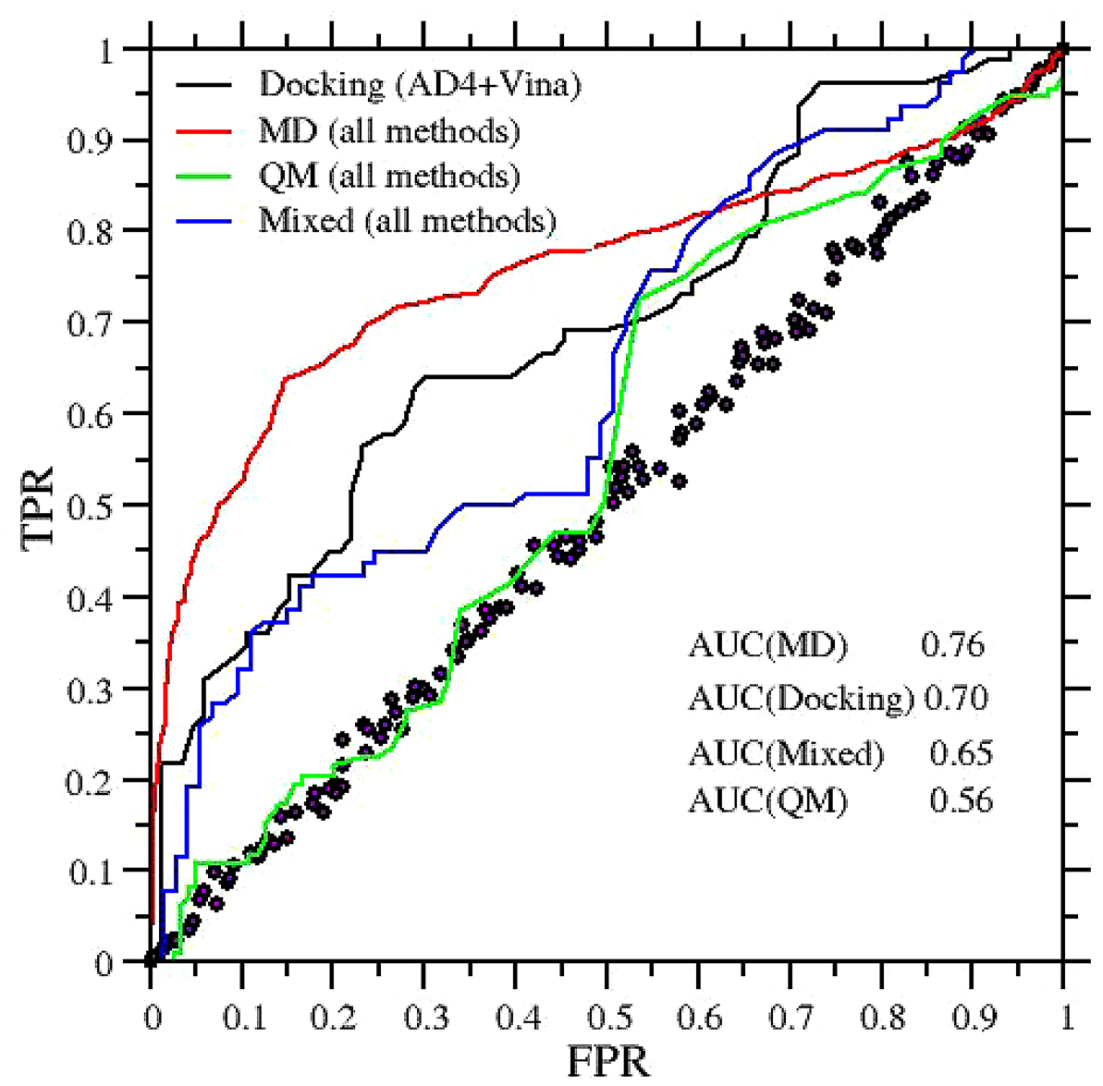
ROC graphs for the various aggregated methodologies used in SAMPL6 SAMPL7 and SAMPL8. The circles represent the random choice (coin flip)

The correlation plots of Fig. 4 translates into the ROC curves reported in the Fig. 5. The area under the ROC curve (AUC) provides a direct measure of how much a methodology is capable of distinguishing between good binders and bad binders. A classification based on a coin flip has an AUC of 0.5. As it can be seen, the best methodology in the SAMPL6-SAMPL8 challenge is MD, with an AUC=0.76. Docking (Autodock4 *and* Vina) yields an AUC of 0.70, in agreement with the mean AUC obtained by docking techniques in the DUD-E ligand-receptor benchmark [20]. Docking performances in the SAMPL challenges, however, are degraded by Vina, the latter showing poor correlation ($\rho =0.05$) and an AUC of 0.55. Autodock4, on the other hand, has an AUC of 0.82, superior to that of the aggregated MD methods. In the SI (directory ROCs in the workspace root directory) we provide the ROC curves of the aggregated methods for the three challenges.

An important point about the ROC curve is that it measures the ability of a method to produce good *relative* instance scores, i.e the ability in ranking the ABFEs of the ligands in the correct order. While ROC graphs are excellent tests for assessing the *precision* (i.e. reproducibility) of a methodology, they tell nothing about the *accuracy*, i.e. how close the prediction is to the actual experimental value. So, for example, a highly inaccurate ($\rm {MAE} \gg 0$) and precise method ($\rho \simeq 1$ and $\tau \simeq 1$) with a correlation plot characterized by a best fitting line with a *positive* slope $\gg 1$ and with arbitrary intercept, yields a ROC graph signaling perfect classification with an AUC$\simeq 1$. A less precise but highly accurate technique (e.g. $\rm {MAE} \simeq 2.5$ kcal/mol), exhibiting a best fitting line with unitary slope and zero intercept, yields and AUC of only 0.9.

## Conclusion

We have tested the Autodock4 program for absolute binding free energy predictions of host-guest systems taken from the recent SAMPL6, SAMPL7 and SAMPL8 challenges. Calculations have been done using the Autodock4 default settings for all cases with no adjustments whatsoever. Using the usual SAMPL metrics based on mean absolute errors and correlation coefficients, we found that Autodock4 performs surprisingly well at predicting binding free energies, surpassing in many instances expensive molecular dynamics or quantum chemistry techniques, yielding on overall an extremely favorable benefit-cost ratio. The Vina1.1.2 docking program was also tested on the SAMPL challenges with less satisfactory results compared to Autodock4

The ROC curves for the aggregated methodologies (MD, QM, Mixed. and Docking) in the SAMPL challenges have shown that the highest AUC are obtained by atomistic molecular dynamics simulations with explicit solvent, followed by Docking (Autodock4 and Vina). Aggregated QM-based or mixed QM/MM are found to be less reliable in ranking absolute binding free energies.

Based on the results reported in our study, a cavalier attitude or excessive skepticism towards docking does not appear to be justified in the computational chemistry community. Given the reported good performances in the SAMPL6-SAMPL8 challenges, and given the limited cost and ease of setup, Autodock4 may provide a valid null (reference) model for future SAMPL challenges.

## Electronic supplementary material

Below is the link to the electronic supplementary material.

Supplementary material 1 (ZIP 20409 kb)
