# Supplementary figures and images for: Binding free energy predictions in host-guest systems using Autodock4. A retrospective analysis on SAMPL6, SAMPL7 and SAMPL8 challenges
Source: J Comput Aided Mol Des. 2021 May 24;35(6):721–9. doi: 10.1007/s10822-021-00388-4 (PMC8141411; doi:10.1007/s10822-021-00388-4)

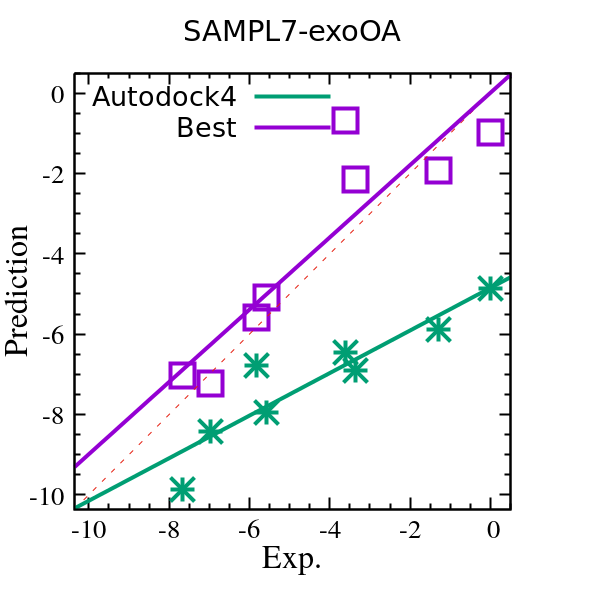

Supplement: Supplementary file 1 — Supplementary material 1 (ZIP 20409 kb) [file 10822_2021_388_MOESM1_ESM.zip › workspace/SAMPL7/exoOA/analysis/SAMPL7-exoOA.png]
